# Pyrosequencing Investigation into the Bacterial Community in Permafrost Soils along the China-Russia Crude Oil Pipeline (CRCOP)

**DOI:** 10.1371/journal.pone.0052730

**Published:** 2012-12-26

**Authors:** Sizhong Yang, Xi Wen, Huijun Jin, Qingbai Wu

**Affiliations:** 1 State Key Laboratory of Frozen Soil Engineering (SKLFSE), Cold and Arid Regions Environmental and Engineering Research Institute (CAREERI), Chinese Academy of Sciences, Lanzhou, China; 2 College of Electrical Engineering, Northwest University for Nationalities, Lanzhou, China; The Roslin Institute, University of Edinburgh, United Kingdom

## Abstract

The China-Russia Crude Oil Pipeline (CRCOP) goes through 441 km permafrost soils in northeastern China. The bioremediation in case of oil spills is a major concern. So far, little is known about the indigenous bacteria inhabiting in the permafrost soils along the pipeline. A pilot 454 pyrosequencing analysis on the communities from four selected sites which possess high environment risk along the CRCOP is herein presented. The results reveal an immense bacterial diversity than previously anticipated. A total of 14448 OTUs with 84834 reads are identified, which could be assigned into 39 different phyla, and 223 families or 386 genera. Only five phyla sustain a mean OTU abundance more than 5% in all the samples, but they altogether account for 85.08% of total reads. *Proteobacteria* accounts for 41.65% of the total OTUs or 45% of the reads across all samples, and its proportion generally increases with soil depth, but OTUs numerically decline. Among *Proteobacteria*, the abundance of *Beta-*, *Alpha-*, *Delta- and Gamma-* subdivisions average to 38.7% (2331 OTUs), 37.5% (2257 OTUs), 10.35% (616 OTUs), and 6.21% (374 OTUs), respectively. *Acidobacteria* (esp. *Acidobacteriaceae*), *Actinobacteria* (esp. *Intrasporangiaceae*), *Bacteroidetes* (esp. *Sphingobacteria* and *Flavobacteria*) and *Chloroflexi* (esp. *Anaerolineaceae*) are also very common, accounting for 8.56% (1237 OTUs), 7.86% (1136 OTUs); 7.35% (1063 OTUs) and 8.27% (1195 OTUs) of total libraries, respectively. The ordination analysis indicates that bacteria communities in the upper active layer cluster together (similar), while bacterial consortia from the lower active layer and permafrost table scatter (less similar). The abundance of *Rhodococcus* (12 OTUs), *Pseudomonas* (71 OTUs) and *Sphingomonas* (87 OTUs) is even less (<0.01%). This effort to profile the background diversity may set the first stage for better evaluating the bacterial dynamics in response to accidental oil spills.

## Introduction

The latitudinal permafrost in northeastern China (0.38×10^6^ km^2^) is mainly distributed in the northern part of the Da and Xiao Xing'an Mountains. The permafrost zone has strong spatial heterogeneity due to the comprehensive geological elements of rugged diverse terrains and complex ecological environments in the northeastern China. As located in the southeast margin of Eurasian cryolithozone, the permafrost body is relatively warmer and is significantly more sensitive to climate change compared to those in Siberia and Alaska. The warming climate has accelerated the degradation of permafrost in past decades, as evidenced by the reduced thickness of permafrost and the increased depths of seasonal thaw, increased ground temperatures, and prolonged northward retreat of the southern limit of permafrost (SLP) [1]. Furthermore, the increasing anthropogenic activities (*e.g.*, agriculture and linear infrastructures) since the 1950s further disturbed and fragmented the permafrost environment.

In 2010, the 953-km-long China-Russia Crude Oil Pipeline (CRCOP) was built with a modified conventional burial construction mode. It crosses 441 km of discontinuous permafrost and 465 km of deep (>1.5 m) seasonally frozen ground [2]. Since then, the CRCOP has been the focus of cold regions environment and engineering practices. For pipelines in cold regions, oil spills normally occur in the early commercial operation due to the differential frost-heave and thaw-settlement, and in the late stages because of cumulative corrosion and aging [3]. Oil spills can cause severe damage to cold ecosystems. For example, at least five accidents in Usinsk in Northern Russia have taken place since 1986, causing very serious adverse environmental impacts [3]. Moreover, the same levels of contamination can heavily damage the cold environments, as the cold ecosystems have adapted to harsh conditions in ways that make them more fragile [4].

Globally, bioremediation is a good choice for remediating petroleum hydrocarbon contaminants because it is more environmentally friendly and cost-effective than other alternative, generally more energy intensive approaches. Many indigenous cold-adapted microbial populations have been observed to be capable to remediate hydrocarbons-contaminated soils at low temperature (*e.g.* [5]–[8]). Microbial degraders could get enriched from less than 0.1% of total bacterial population in pristine environments to up to 100% of the population [9]. Hydrocarbons ranging from C_10_ to C_26_ and aromatics of low molecular weight can be readily degraded, while more complex molecular structures are generally more resistant to biodegradation [10].

Bioremediation research in China has been enhanced in recent years. Microbial degraders were isolated from polluted soils in Liaoyang, Dagang [11]–[13], Shengli oil fields [14] and wetlands in the Liaodong Bay of China [15]. Some studies investigated the soil microbial diversity in Huabei, Dagang Oil fields in North China [16], [17], and community changes in response to contaminants in Changqing oil field [18], Daqing [19] and Yellow River Delta [20]. Some pilot experiments were also carried out on oil field soils at the bench scale [12], [21] and field scale [22]. In addition, bioaugmentation technique was also tested in Dalian Bay recently [23]. Generally, almost all of these works focus on temperate oil fields where spills came out after long-time exploration.

So far, little is known about hydrocarbon degrading bacteria in the permafrost soils in the northeastern China. According to the environmental impact statement, high risks for oil leakage should be anticipated in segments along the CRCOP with the complicated engineering geology, particularly when the pipeline crosses the natural wetland reserves or important water supply sources [24]. The bioremediation of hydrocarbon pollutants is therefore very practical and necessary. This pipeline provides an opportunity to investigate the microbial degradation in case of oil spills. Current research on this subject is still in infancy. The knowledge about specific indigenous microbes, genes, and enzymes involved in hydrocarbon biodegradation within the permafrost environments along the pipeline is still scarce.

Considerable numbers and biodiversity of bacteria has been found in permafrost soils [25]. A global survey of 16S rRNA libraries indicated that *Proteobacteria* and *Acidobacteria* comprised roughly 40% and 20% of the communities, and *Chloroflexi* and *Firmicutes* were also abundant (>5% of the community) in a wide range of soil environments [26]. Molecular techniques revealed the dominance of *Gammaproteobacteria* (esp. *Xanthomonadaceae*, 75–84%) and *Actinobacteria* (39–57%) in Siberian permafrost, and Gram-positives (up to 45%) and *Proteobacteria* (up to 25%) in Antarctic permafrost [27], [28]. Canadian Arctic permafrost is dominated by members of *Proteobacteria*, *Actinomycetes*, *Firmicutes*, *Planctomyces*, CFB, and *Gemmatimonadetes* [25]. Some groups are very important to carbon turnover. The dominant methane-oxidizing *Proteobacteria* (MOP) is affiliated with the *Gamma-* (type I) and *Alpha- proteobacteria* (type II) [27].

Conventionally, the biodiversity of permafrost microbes can be rapidly profiled by the DNA fingerprint methods, but the patterns of abundance and co-occurrence depend on the resolution of analytical methods [29]. Recently, pyrosequencing showed promise to capture the microbial taxa especially the low-abundant species. It can eliminate the laborious step of producing clone libraries and generate large number of sequences in a single run [30]. This work reported our pioneer research in high-throughput sequencing, and the results reveal an immense bacterial diversity in the active layer and permafrost table at four sites along the CRCOP pipeline. More importantly, this work may help explore the natural bioremedial consortia in the permafrost soils along the CRCOP.

## Research Sites

The permafrost along the CRCOP is in the south margin of the latitudinal permafrost in East Asia [2]. The pipeline crosses both seasonally frozen ground and permafrost regions, where discontinuous, sporadic and isolated patches of permafrost were developed on the basis of varied terrains, soil textures, water contents, slope aspects and vegetative coverage. The heterogeneous conditions distinguished the CRCOP from the Alyeska Pipeline system and Norman Wells Pipeline. The complex permafrost conditions have greatly complicated the design and construction of the CRCOP pipeline [2]. According to the environmental assessment, high-risk of spills were considered in the 157 sections where pipeline cross the transition area with different permafrost conditions [24].

**Figure 1:**
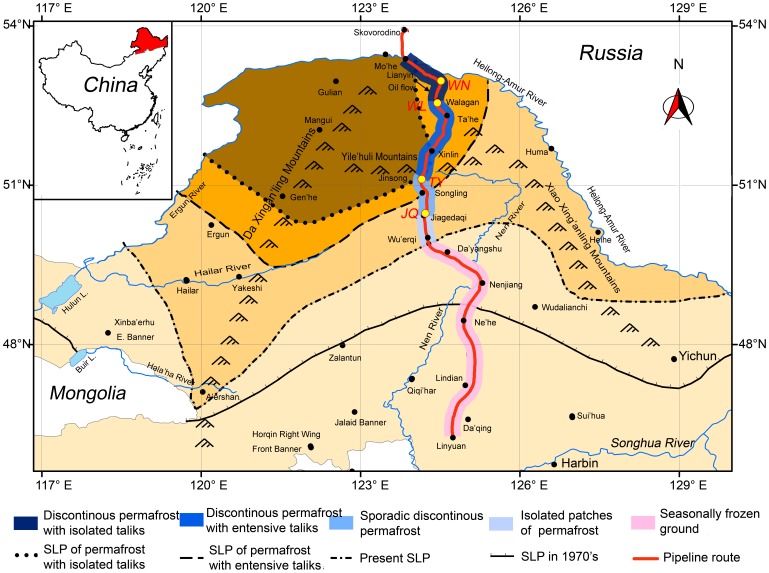
Schematic map showing the sampling sites along the China-Russia Crude Oil Pipeline route (after Jin *et al.* [**2**]). WN: Walagan north; WL: Walagan; TY: Tayuan; JQ: Jiagedaqi; SLP: Southern limit of permafrost.

In this study, four research sites were selected along the pipeline, which potentially represent four permafrost subzones prone to oil spills (Figure 1, referring details to Jin *et al.* [2]). All sites stand on permafrost-affected bogs, but differ in area extent, soil temperature and permafrost thickness. Microbial soil samples were collected during the pipeline trench excavation in winter of 2009. For each profile, triplicate samples were retrieved from layers 1) the upper active layer (normally 30–40 cm, below the modern grass rhizosphere), 2) the lower (deep) active layers (depth depending on the sites), and 3) the permafrost table. The soil samples were collected in the state-owned land which is open for scientific research. No specified permissions are required for these sampling sites, which are not natural reserve and did not involve endangered or protected species.

## Methods

### DNA extraction, PCR and pyrosequencing

Soil DNA was extracted using commercial Power Soil DNA Isolation Kit (MOBIO, USA) according to the instruction. The triplicate DNA extracts for each layer were pooled prior to downstream manipulation. Universal bacterial primer set 8F (5′-3′ GAGTTTGATCCTGGCTCAG) and 533R (5′-3′ TTACCGCGGCTGCTGGCAC) covering V1–V3 regions of SSU were synthesized by Shanghai Majorbio Bio-pharm Technology Co., Ltd. (Majorbio as below). Different barcode sequences were added at the 5′ end of the forward primer for multiplexed pyrosequencing. PCR were carried out in a 20 µL reaction volumes containing 0.5 ul DNA template, 250 µM dNTPs, 0.1 µM of each primer and 2.5 U FastPfu Polymerase (Applied Biosystems) in the appropriate 5× FastPfu Buffer and de-ionized ultrapure water. The protocol was optimized with low cycles for better accuracy and reliability of the subsequent data analysis. The PCR condition were initial denaturation at 95°C for 2 min, followed by 25 cycles of denaturation at 94°C for 30 s, annealing at 55°C for 30 s and extension at 72°C for 30 s, with a final extension phase at 72°C for 5 min. PCR products (3 µL) were checked on a 2% agarose gel. PCR products were purified using MiniElute PCR purification kit (Qiagen) and quantified using the GeneQuant pro system. Samples were then pooled at equal concentrations. Parallel tagged sequencing was performed using a Roche 454 GS FLX in Majorbio.

### Bioinformatic analysis

Data preprocessing was performed mainly upon software of mothur [31]. The raw sequence was trimmed off the standard primers and barcodes, assembled the reads to contigs. Sequences less than 150 bp in length and greater than 3% low quality bases (quality score <27) were removed. The chimeric sequences were also excluded by the chimera.uchime command with default parameters. These valid sequences were finally trimmed to 300 bp and then aligned with needleman algorithm and clustered with the bacterial SILVA database (SILVA 108). The candidate sequences were assigned to the taxonomy with classify.seqs command (Bayesian approach). And the dist.seqs command generated the distance matrix between aligned DNA sequences. Gap comparisons and terminal gaps were handled with the method option of calc = onegap and countends = T. Then, these sequences were clustered to OTUs (operational taxonomic units) at 97% sequence identity by using mothur (furthest neighbor method) and chopseq (Majorbio). Rarefaction analysis was performed by mothur and plot-rarefaction (Majorbio). From these, the Shannon diversities and the Chao1 richness estimations were calculated by mothur. The weighted UniFrac distance was used to quantify differences in community composition. Heatmap figure and Venn diagrams were implemented by *R* packages pheatmap [32] and VennDiagram [33], respectively. In addition, weighted principal component analysis (PCA) and Nonmetric Multidimensional Scaling (NMDS) diagrams were generated by using *R* package vegan [34] to demonstrate the clustering of different samples. The sequences for this article have been deposited in NCBI SRA under the accession number SRA057910.

## Results

**Figure 2:**
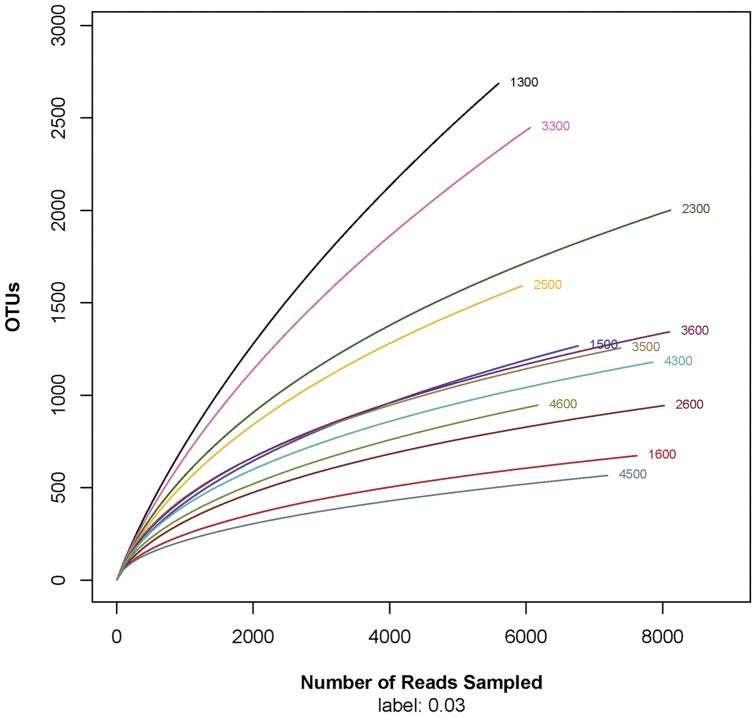
Rarefaction analysis of the different samples. Rarefaction curves of OTUs clustered at 97% phylotype similarity level. The sample labels beginning with numeric 1, 2, 3 and 4 correspond to the sampling locations of Walagan (WL), Walagan north (WN), Tayuan (TY) and Jiagedaqi (JQ). The second digit (3, 5, 6) in the labels represents the upper, the lower active layer and the permafrost table, respectively.

A total of 84834 reads and 14448 OTUs were obtained from the 12 samples through 454 pyrosequencing analysis. Each library contains 5600 to 8117 reads, with different phylogenetic OTUs ranging from 566 to 2687. The rarefaction curves tend to approach the saturation plateau except in the samples of 1300 and 3300 (Figure 2). The Good's coverage index reveals that 90% to 96% of the species were obtained in eight samples, and 85–87% for samples of 2500 and 2300, while there are still some fraction of species diversity remains to be discovered in the samples of 1300 (68%) and 3300 (74%) respectively. The samples from upper active layer (*e.g.*, the samples of 1300, 2300, 3300 and 4300) were plotted in the upper part in Figure 2. This rarefaction curve indicates a large variation in the total number of OTUs in different samples, but the sequence coverage is still sufficient to capture the diversity of the bacterial communities, whereas the OTUs density is larger in the upper layer than the lower parts.

### Taxonomic composition

Out of the twelve samples they comprised of different numbers of OTUs and OTU abundances. Sequences that could not be classified into any known group are assigned as *No_Rank*. These bacterial OTUs can be assigned into 39 different phyla, 223 families or 386 genera. Thirteen different phyla out of the 39 total phylotypes are common to the whole 12 libraries, which occupy 97.53%, 96.38%, 95.32%, 93.92% of the total reads in the libraries of Walagan (WL), Walagan North (WN), Tayuan (TY) and Jiagedaqi (JQ), respectively. Only five phyla comprise mean OTUs abundance more than 5% in each sample, but they jointly hold 85.08% of the total reads. *Proteobacteria* is the most abundant division (Figure 2), comprising approximately 41.6% (6018) OTUs and 45.08% (38196) reads across all samples, whereas the members from *Acidobacteria* (8.56%, 1237 OTUs), *Actinobacteria* (7.86%, 1136 OTUs), *Bacteroidetes* (7.35%, 1063 OTUs) and *Chloroflexi* (8.27%, 1195 OTUs) make up 8.20% (6955 reads), 8.16% (6922 reads), 6.38% (5413 reads) and 5.10% (4325 reads) of the whole libraries, respectively. The average reads of *No_rank* group accounts for 2.78%, but fluctuates in different sites and depths. There are also a certain proportion of members of *Firmicutes* (10.37%, 8801 reads; 4.94%, 715 OTUs), *Planctomycetes* (0.95%, 782 reads; 3.80%, 549 OTUs), *Verrucomicrobia* (1.65%, 1401 reads; 1.75%, 253 OTUs) and *Nitrospirae* (1.34%, 1136 reads; 0.82%, 119 OTUs). The other lineages represent much smaller fraction (ca. 7%) of the bacterial communities.

At family level, there are 19 lineages among the total 223 families exist in all samples, but these subgroups account for 71.86%, 70.28%, 80.25% and 80.68% of the total reads in the four research sites, respectively. The top 5 families, by descending read abundance, are *No_Rank* (32.35%), *Oxalobacteraceae* (15.86%), *Comamonadaceae* (9.90%), *Flavobacteriaceae* (2.37%) and *Nitrosomonadaceae* (2.09%). According to OTUs, the top 5 phylotypes are *Comamonadaceae* (4.39%, 635 OTUs), *Oxalobacteraceae* (3.70%, 535 OTUs), *Sphingomonadaceae* (3.17%, 459 OTUs), *Caulobacteraceae* (2.42%, 350 OTUs), and *Chitinophagaceae* (1.89%, 274 OTUs).

**Figure 3:**
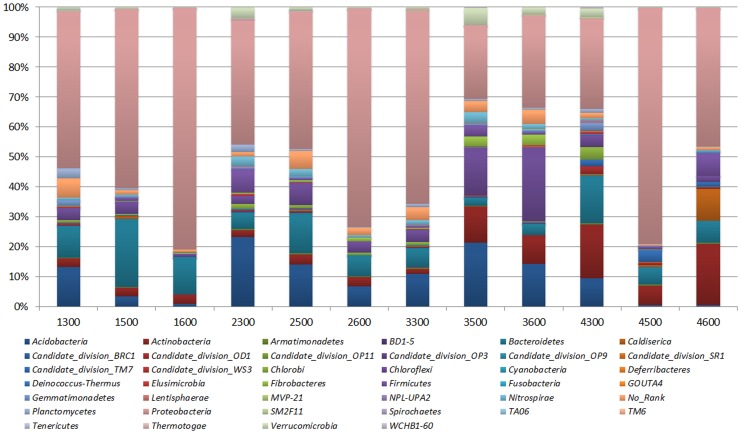
Bacterial composition of the different communities at the phylum level. Relative read abundance of different bacterial phyla within the different communities. Sequences that could not be classified into any known group were assigned as *No_Rank*.

Generally, each site contains approximately the similar phyla, and all have shown higher phylotypic richness in the upper active layer than the lower layers (Table 1). For example, the WL profile descends from 31 bacterial phyla in the upper active layer (1300), to 24 in the lower active layer (1500), and till to 14 lineages in the permafrost table (1600). Similar changes can be found in WN. The TY and JQ profiles, however, show relatively lower richness in the lower active layer (70–80 cm). In addition, these bacterial consortia differ vertically in the relative proportions of different phyla in the whole libraries. A striking feature is that the portion of the dominant *Proteobacteria* readily increases but the number of OTUs decrease with depth in three research sites, especially in WN and WL which are located in the northern part of the pipeline route, while JQ displays its highest peak in the lower active layers. On the other hand, the TY profile demonstrates a generally descending trend (Figure 3). The second predominant phylum of *Acidobacteria* shows an increasing trend in WN, WL and JQ, but declines in TY. The division of *Bacteroidetes* sustains relatively similar read abundance in the upper active layer and permafrost table, but a peak occurs in the deep active layer of the WL (10% vs 6%) and WN (22% vs 13%), that is unlike the gradual decrease trend in profiles of TY and JQ. The other phyla show unsystematic vertical variations in comparison to the *Proteobacteria* and *Acidobacteria*.

**Table 1:** Vertical variations of bacterial phyla in the four research profiles.

Horizon	WN		WL		TY		JQ	
	Depth(cm)	phyla	Depth(cm)	phyla	Depth(cm)	phyla	Depth(cm)	phyla
Upper active layer	30–40	32	30–40	31	30–40	31	30–40	30
Lower active layer	70–80	26	70–80	24	80–90	27	70–80	26
Permafrost table	130–140	23	150–160	14	120–130	29	140–150	29
Total observed		33		33		34		35

The members of *Betaproteobacteria* dominate the *Proteobacteria* phylum, occupy 16.13% (2331 OTUs) of total, but take over 34.03% of the total reads (ranging from 17.63–75.58%). The subdivision of *Alphaproteobacteria* sustains 15.62% (2257) of the total OTUs, and only an average of 6.10% of total reads but showing high variability (0.64%–26.74%). *Gammaproteobacteria* and *Deltaproteobacteria* make up 2.58% (374 OTUs) and 4.26% (616 OTUs) in total phylotypes, and have 5.97% and 5.56% reads in the total, respectively. The *Epsilonproteobacteria* only contains 8 OTUs (0.05%) and a very small fraction (0.31%) in total reads. Statistically, the members of *Betaproteobacteria* contribute 38.73% to the total *Proteobacteria* reads, and *Proteobacteria* fluctuation is strongly correlated with the *Betaproteobacteria* (coefficient is 0.78) across all samples, particularly in the TY and JQ samples. At the permafrost table of WL (1600) and WN (2600), high abundant *Alphaproteobacteria* (34.94%) and *Deltaproteobacteria* (23.12%) further raise the read abundance of *Proteobacteria*. Among the *Bacteroidetes*, members of *Sphingobacteria* and *Flavobacteria* are most abundant, making up 3.62% and 2.41% accordingly in total reads. But vertically, *Bacteroidetes* is dominated by members of *Chitinophagaceae* (1.55%) in the upper layer and *Flavobacteriaceae* (3.77%) at the permafrost table. The members of *Actinobacteria* are less abundant in the upper active layer compared with permafrost table. Among *Actinobacteria*, the most common group is *Intrasporangiaceae* with 1.24% reads in the total libraries of the lower active layer and *Micrococcaceae* (1.09%) at the permafrost table, respectively.

### Bacterial community variation with sites and depths

**Figure 4:**
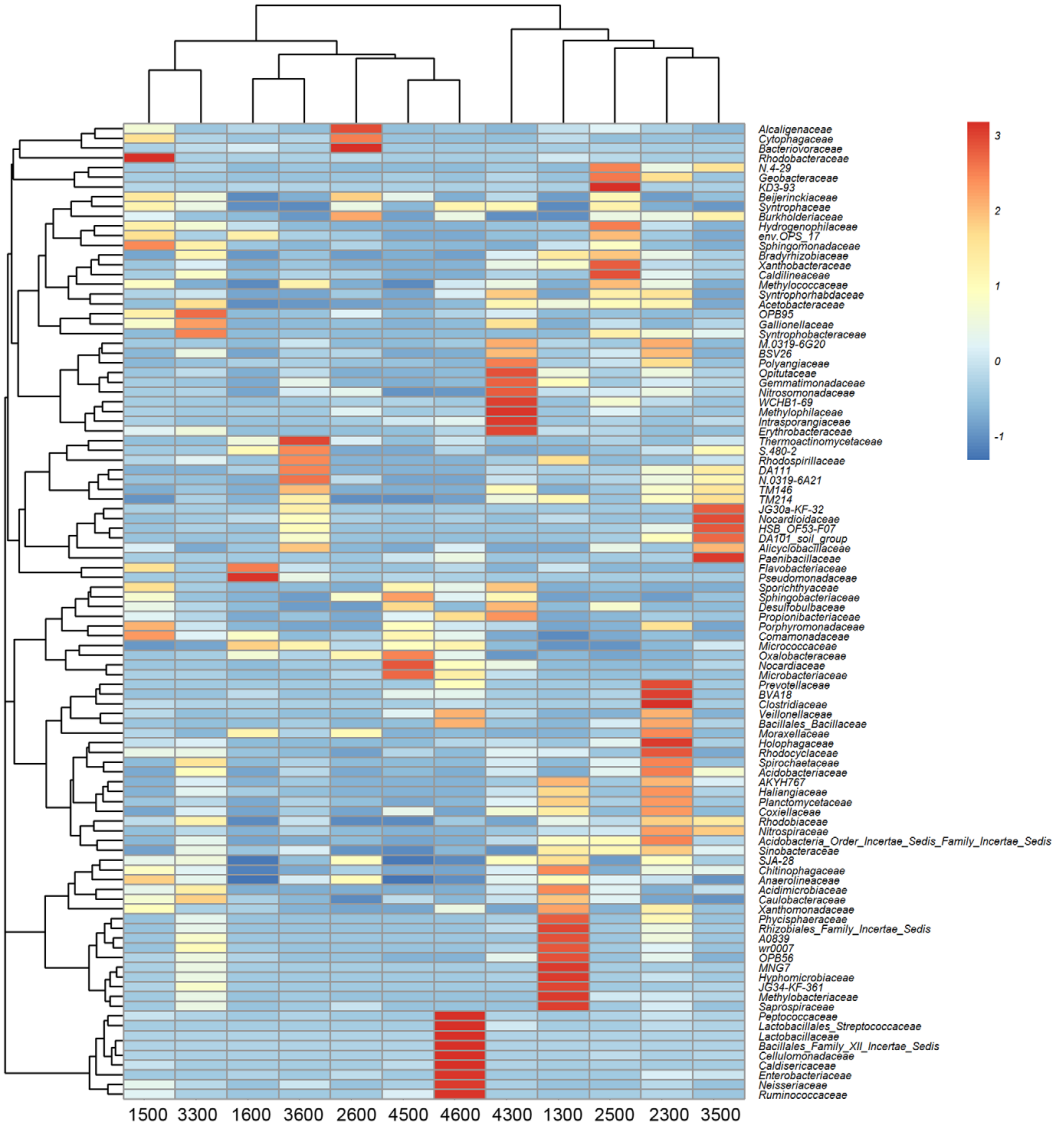
Bacterial distribution of the top 100 abundant families among the twelve samples. Double hierarchical dendrogram shows the bacterial distribution. The bacterial phylogenetic tree was calculated using the neighbor-joining method and the relationship among samples was determined by Bray-Curtis distance and the complete clustering method. The heatmap plot depicts the relative percentage of each bacterial family (variables clustering on the vertical-axis) within each sample (horizon-axis clustering). The relative values for bacterial family are indicated by color intensity with the legend indicated at the top right corner.

**Figure 5:**
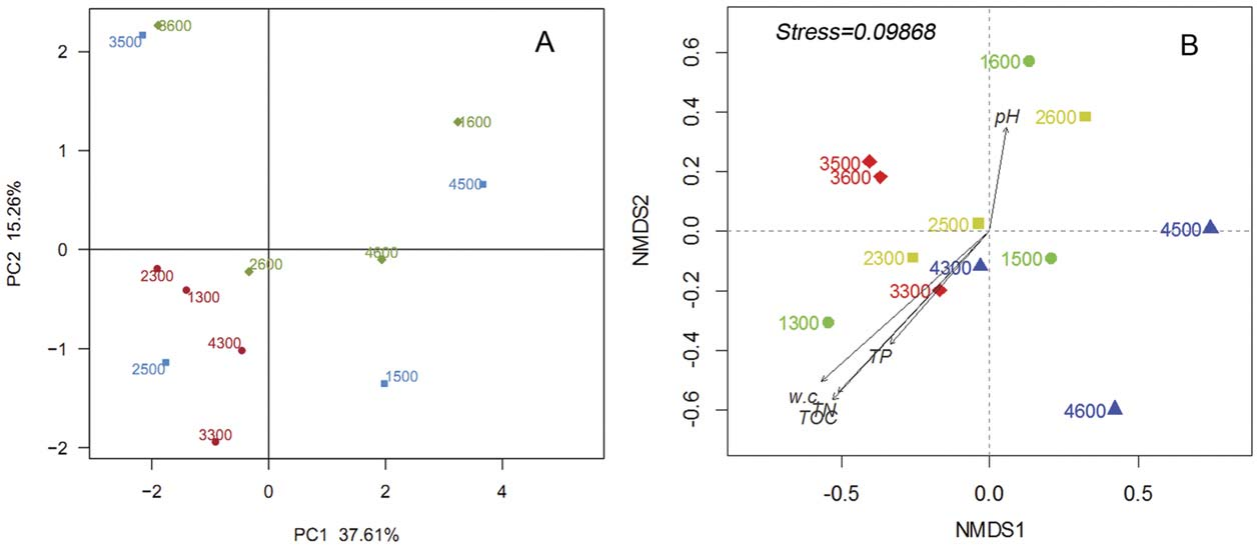
Sample sorting analysis. (A) Scatter plot of PCA-score showing similarity of the 12 bacterial communities based on Unifrac distance. Principal components (PCs) 1 and 2 explained 37.61% and 15.26% of the variance, respectively. (B) NMDs showing the difference of bacterial communities according to Bray-Curtis distance. TOC: total organic carbon, TN: total nitrogen, TP: total phosphorous, and w.c.: absolute water content (per gram dry soil).

The hierarchical heatmap (Figure 4) is based on the top 100 abundant bacterial community at family level, which generally indicates two groups. One is mainly composed of the upper layer samples (1300, 2300, 4300); the other is chiefly clustered by the deep samples (1500, 1600, 2600, 3600, 4500, 4600). According to the heatmap, high similarity of the communities could be found in the same profile (*e.g.*, lower active layer (4500) and permafrost table (4600) in JQ), or different sites (*e.g.*, permafrost table samples of 1600 and 3600), or different layers in different sites (*e.g.*, WN (2300) and TY (3500)). The principal component analysis (PCA) (Figure 5A) indicates that the upper active layer communities group together in the bottom left of the graph along PC1, whereas the communities in the lower active layer and the permafrost table scatter in different parts in the plot. Clearly, the upper active layer clusters are closer (similar) than those in the lower active layer (1500, 3500 and 4500) and the permafrost table (1600, 2600, 3600 and 4600). The PCA analysis agrees with the heatmap to indicate the far dissimilarity between 1500 and 3500, or high similarity between 2600 and 4600 libraries. The NMDS analysis based on the Bray-Curtis distance (Figure 5B), also confirms the more similar bacterial communities in the upper active layers than the other layers. The direction and position of the environmental factors, calculated with the envfit function in the *R* vegan package [34], suggest the bacterial community in the upper active layers is somewhat related to the environmental parameters of total organic carbon (TOC), total phosphorus (TP) and nitrogen (TN), and water content. However, the correlation does not reach statistical significance.

**Figure 6:**
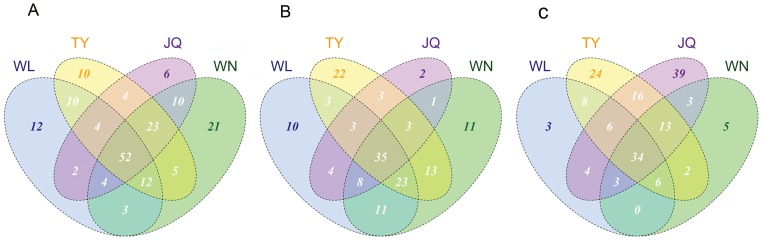
Venn diagram showing the unique and shared OTUs (3% distance level). (A) Upper active layer (30–40 cm, including samples of 1300, 2300, 3300 and 4300), (B) Lower active layer (70–80 cm, including 1500, 2500, 3500 and 4500) and (C), Permafrost table (below 130 cm, including 1600, 2600, 3600 and 4600) libraries. WL: Walagan, WN: Walagan North, TY: Tayuan, JQ: Jiagedaqi.

The shared communities at different layers are further determined via the Venn diagram (Figure 6). In the upper active layer below the modern grass rhizosphere, a total of 52 families are shared by the four research sites (Figure 6A), accounting for 29.21% of the total 178 bacterial taxa in this layer. The top 10 abundant families shared by the four sites, according to the relative read abundance in the top 10, are unclassified *No_Rank* groups (10969 reads, holding 39.69% of the total), *Betaproteobacteria* (2792 reads), *Alphaproteobacteria* (772 reads, esp. 437 *Xanthobacteraceae* and 271 *Bradyrhizobiaceae*), *Bacteroidetes* (428 reads, esp. *Chitinophagaceae*, 428 reads), *Gemmatimonadetes* (374 reads, mostly *Gemmatimonadaceae*), *Chloroflexi* (372 reads, esp. *Anaerolineaceae*) and *Planctomycetes* (317 reads, esp. *Planctomycetaceae*). Among the *Betaproteobacteria*, the dominant members are assigned into *Comamonadaceae* (1530 reads), *Nitrosomonadaceae* (1007 reads), and *Rhodocyclaceae* (255 reads).

In the lower active layer, a total of 152 families of bacteria are identified, 35 of them (23.02%) are common in all the four research sites (Figure 6B). Of the top 10 families, the *Betaproteobacteria* with average 9315 reads is the first dominant group, and it is mainly composed by members from *Oxalobacteraceae* (5400 reads, 19.79%) and *Comamonadaceae* (3915 reads, 14.35%). The *No_Rank* group, having 8514 reads (31.20%), is also very common in this layer. Additionally, the libraries at the permafrost table, sharing 34 families, occupy 20.4% of the total 166 discovered taxa (Figure 6C). *Betaproteobacteria* is still predominant, taking up 33.68% of the total read abundance, which consists largely of members from *Oxalobacteraceae* (6621 reads, 22.13%) and *Comamonadaceae* (2957 reads, 9.88%). The *No_Rank* group is also abundant, containing 26.61% (7961 reads in total). Particularly, highly abundant *Gammaproteobacteria* is detected at the permafrost table, comprising a large proportion of *Pseudomonadaceae* (2850 reads, 9.53%) and *Moraxellaceae* (850 reads, 2.84%). Throughout the whole profiles, the bacterial communities in the upper layer are more diverse and shared more taxa than the other two layers.

## Discussion

The permafrost along the CRCOP is overall warmer than that in Siberian Arctic, or contains higher carbon and water contents than that of the Tibetan Plateau, which may present a unique permafrost eco-niche in China. The 454 pyrosequencing in this study enable us to quickly capture both high and low-abundance phylotypes in a single run, to circumvent potential bias that conventional fingerprints and cloning procedures might introduce, and thus provide a different picture which can greatly improve our knowledge about the indigenous bacteria in permafrost soils along the CRCOP.

### Biodiversity background

Based on our results, the permafrost soils along the CRCOP sustain an immense diversity of bacteria than previously expected. The predominant phyla are *Proteobacteria* (esp. *Beta-* and *Alpha-* subdivision), *Acidobacteria*, *Bacteroidetes*, *Actinobacteria*, *Chloroflexi*, *Firmicutes*, and *Verrucomicrobia*. The *Chloroflexi* sustains relatively high OTUs (8.27%), but lower read abundance (5.10%), while *Firmicutes* has low OTUs fraction (4.95%) but high portion of reads (10.37%). The typical peat-inhabiting *Betaproteobacteria* from acidic permafrost wetland are acidotolerant bacteria and can utilize various carbon substrates and fix N_2_ [35]. From this point, the acidic nature of the permafrost soil in this study is likely favorable for the members of *Betaproteobacteria*. This is unlike the pyrosequencing result of the arctic permafrost wetlands where the most common members are from *Chloroflexi*, *Acidobacteria*, and *Actinobacteria* (esp. *Intrasporangiaceae* and *Rubrobacteraceae*) [36]. The CFB groups with low members in this research are coincidence to those from the Siberia and Antarctic permafrost [28]. Recently, cultured representatives of *Acidobacteria* from cold peat bogs were found as acidophilic chemo-organotrophs that can grow at pH values between 3.0 and 6.5–7.5, and peat-inhabiting members of *Actinobacteria*, together with *Verrucomicrobia*, are less abundant but numerically significant groups of sequences in clone libraries in peat soil [35].

Surprisingly, the *No-Rank* read abundance accounts for a large proportion (>30% in average) in this study, a certain extent of candidate divisions are also detected. Similarly, unclassified bacteria could make up nearly 30% of clones in Arctic tundra soils, which probably play a significant yet unknown or less understood ecological role [28]. On the other hand, the 454 dataset offers a large suite of low-abundance communities with average fractions <0.1%, but they occupy 69% and 82% of total bacterial families and genus observed, accordingly. Similarly, low-abundance populations were revealed by pyrosequencing to greatly contribute to the phylogenetic diversity in deep water of the North Atlantic and hydrothermal vents [37]. Moreover, the members of *Planctomycetes* with low proportion are commonly observed in this study, were previously reported to be strongly underrepresented in the clone library from cold peatlands [35]. Since little is known about the global distribution of the rare populations, it is not yet possible to know whether they represent specific biogeographical distributions of bacterial taxa, or functional selection by particular permafrost environments.

Our dataset also indicated higher phylotypic richness is present in the upper active layer than lower layers, probably because the upper layer is in the oxic- anoxic interface, *i.e.*, from the region of highest biological activity. The abundance of *Proteobacteria* generally increases with soil depth, mostly attributable to the subdivision of *Betaproteobacteria*. This is different from the result that *Gammaproteobacteria* members dominated in some anaerobic soils [38]. Another abundant group of *Acidobacteria*, often found in low pH soils [29], its abundance also tends to increase with depth in this study. Such increasing abundance was also identified in Siberia permafrost soils [25]. Generally, the microbial community difference is due to variations in permafrost environment [29]. For examples, the Siberian bacterial diversity in permafrost lower than the active layer is attributed to the low water availability, extremely low rates of nutrient exchange, and prolonged subfreezing temperature [25]. Soil redox conditions in permafrost were assumed to shape different structure of bacterial communities [39]. In addition, vegetation also exerts influences on bacterial community structure, *e.g.* in Alaskan soils [40]. In this study, the shifts in bacterial community structure are related, but not statistically to the degree of soil saturation, and the contents of TOC, TN and TP in the permafrost profile.

### Potential degraders

Many of the bacteria capable of degrading oil are Gram negative and rod-shaped. Alkane-degrading bacteria from cold soils are frequently assigned to the Gram-positive genus *Rhodococcus* or the Gram-negative genus *Pseudomonas*; Aromatic-degrading bacteria from polar soils are typically classified into the Gram-negative bacterial genera *Pseudomonas* or *Sphingomonas* [10]. Except for the major groups, the biodegradation of alkanes were found correlated with groups related to denitrification, sulfate reduction from some years [41]. The *Pseudomonas* can degrade a narrower range of aromatic substrates than *Sphingomonas* spp. [10]. And, *Acinetobacter* from *Gammaproteobacteria*, contribute to the mineralization of aromatic compounds [42]. In our samples, *Pseudomonas* genus mostly exists in the deep active layer and permafrost table, with total OTUs fraction ca. 0.5%, however, the read abundance only occupies 0.3% (257 reads) of the total libraries. The *Rhodococcus* is a rare group across the 12 libraries, and only 12 OTUs are totally identified at genus level. *Sphingomonas* genus contains 87 OTUs but only 32 reads in the twelve samples. Among them, the potential degraders are expected to be explored. Some species in *Flavobacterium* genus capable of degrading pentachlorophenol [43], [44], are also observed in our samples, for example, 5 reads of *Achromobacter* and 1508 reads of *Flavobacterium*.

Populations of hydrocarbon-degraders normally constitute less than 1% of the indigenous microbial communities [45], which could be echoed by the groups less than 1% in this study. However, hydrocarbon contaminants can greatly shift both in quantity and composition [9]. Numbers of bacterial degraders can become elevated in contaminated soils to reach 10% to even 100% of the community [45], but the overall microbial diversity generally declines [10]. In Antarctic, hydrocarbon contamination could lead to enrichment of degrader of *Proteobacteria* (esp. genera *Pseudomonas*, *Sphingomonas* and *Variovorax*), and some *Actinobacteria* [46]. In the temperate Yellow River Delta in China, *Alpha*-, *Beta-* and some unknown *Gamma- proteobacteria* were enriched whereas *Deltaproteobacteria*, *Firmicutes*, *Actinobacteria*, *Acidobacteria* and *Planctomycetes* members were depleted by heavy crude spills [20]. The *Gammaproteobacteria* members could be enriched up to 30% of the total clone libraries observed in the seasonally frozen ground in Daqing, Northeast China, particularly members from Acinetobacter , then *Arcobacter*, *Firmicutes*, *Pseudomonas*, and *Sulfurospirillum* [47]. The strong shift towards the *Gammaproteobacteria* was also found in the Arctic sea-ice [48]. At present, the dynamic response of permafrost bacteria to the crude oil transported in the pipeline remains little known, but it would be the future focus.

The potential bioremediation can be affected by many factors. The number of active biodegraders certainly matters. Currently, total microbial numbers in these samples are generally at the magnitude of 10^7^ for indigenous consortia, and could quickly decline to 10^3^∼10^4^ after 8 weeks in a lab contamination experiment (data not shown). A long period of incubation showed that degraders who are low or below detection limits in pristine polar soils could be enriched to more than 10^5^·g^−1^ in contaminated soils in both surface and subsurface layers [49], [50]. Other important factors include the composition of the oil, availability of nutrients and oxygen, water content, temperature, pH and salinity in cold regions [9]. Importantly, most microbial oil degradation strongly depends on the availability of oxygen, and nitrate or sulfate serving as a terminal electron acceptor [7]. In addition, temperature-water interaction can impact the bioremediation effectiveness due to their limitation to the oxygen availability in permafrost, especially during seasonal transition periods [10]. However, microbial degradation does not proceed linearly over time, it generally slows as the more readily degradable components are used up, leaving behind the more recalcitrant components [49].

### Potential in carbon turnover

In the Da Xing'an Mountains, about 12% surface is covered by permafrost-affected-peat bogs with average peat layer of 0.5–1 m [51]. The total soil organic carbon content averages to 30% in the active layer and 10% in the vicinity of the permafrost table in this study (data not shown). The biogenic carbon decomposition should be particularly considered when this permafrost unit degraded rapidly. Our data showed a high abundance of type II MOP in the active layer, and the type I *Gammaproteobacterial* methanotrophs at the permafrost table. Among the type II MOP, the major read abundant groups shift from *Xanthobacteraceae* (1.58%) and *Bradyrhizobiaceae* (0.98%) in the upper of active layer, to *Sphingobacteriaceae* (0.62%) in the deep active layer and to *Sphingomonadaceae* (0.27%) at the permafrost table. This pattern differs from those in cold peatlands where the type II methanotrophs and methylotrophs mainly comprise the members from families of *Methylocystaceae* and *Beijerinckiaceae* [35]. On the other hand, most members of type I jump to a peak value of 12.36% at the permafrost table. Most members belong to the families of *Pseudomonadaceae* (9.53%) and *Moraxellaceae* (2.84%).

Obviously, the type II members generally decrease with depth, whereas the type I MOP is higher than type II only at the permafrost table (12.43% and 1.12%, respectively). The two types of MOP differ in carbon assimilation pathway. Due to this reason, the dynamic of the two MOP groups in the degrading permafrost environments could determine the major players and related major metabolic patterns in different sites and soil layers. Recent thawing of Alaskan permafrost soil was shown to have caused a rapid and dynamic shift of microbial communities involved in cycling of carbon and nitrogen. The type II methanotrophs significantly increased in abundance after thaw, methane previously accumulated in permafrost is released during the thawing process and subsequently consumed by methanotrophic bacteria [52]. The climate warming has accelerated the permafrost degradation and drying of permafrost wetlands in Northeast China [1]. While thawing, the trapped *Gammaproteobacterial* MOP is likely to be activated, and trapped organic matter may become more accessible to microbial degradation and subsequently enhance greenhouse gas emissions.

## Conclusion

The 454 pyrosequencing reveals an immense bacterial community in the permafrost soils along the CRCOP in the northern part of Northeast China. These members could be assigned into 39 different phyla, and 223 families or 386 genera. Among them, only five phyla comprise average OTUs abundance more than 5% across all samples, but they altogether account for 85.08% of total reads. *Proteobacteria* (mostly *Betaproteobacteria* and *Alphaproteobacteria*) is predominant, making up 41.65% (6018 OTUs) of the total OTUs identified, followed by *Acidobacteria* (8.56%, 1237 OTUs; esp. *Acidobacteriaceae*), *Chloroflexi* (8.27%, 1195 OTUs; esp. *Anaerolineaceae*), *Actinobacteria* (7.86%, 1136 OTUs; esp. *Intrasporangiaceae*), *Bacteroidetes* (7.35%, 1063 OTUs; esp. *Sphingobacteria* and *Flavobacteria*). The potential biodegraders are anticipated to be found within the genera of *Rhodococcus*, *Pseudomonas* and *Sphingomonas* which are less than 0.01% in the indigenous bacterial communities. Moreover, the low abundant groups (<0.1%) could account for 69% and 82% of total numbers of families and genus observed in this research. Besides, the communities in the upper active layer are relatively more similar to each other than those in the lower active layer and in the permafrost table. The former one seems to be more correlated with the soil TOC, TN, TP, and soil water contents. This study is somewhat weakened in the strength of its conclusions because the lack of biological replicates. However, the results from this study still profile the background bacterial communities among which potential hydrocarbon degraders could be further discovered.
