# Correction: Pyrosequencing Investigation into the Bacterial Community in Permafrost Soils along the China-Russia Crude Oil Pipeline (CRCOP)

**DOI:** 10.1371/annotation/622db7c2-5ca8-402a-b4f2-666dc31172ab

**Published:** 2013-11-19

**Authors:** Sizhong Yang, Xi Wen, Huijun Jin, Qingbai Wu

The number of the grant from the National Natural Science Foundation of China is incorrect. The correct grant number is: 40901044. 

